# A Multimodal Representation Learning Framework for Molecular Graph and NMR Spectrum Alignment

**DOI:** 10.3390/e28050532

**Published:** 2026-05-07

**Authors:** Xiao Li, Xun Wang, Zhong-Ming Liu, Jin-Biao Liu, Xin Huang

**Affiliations:** 1 School of Artificial Intelligence, Jiangxi Normal University, Nanchang 330022, China; 202441600125@jxnu.edu.cn (X.L.); 202226703019@jxnu.edu.cn (Z.-M.L.); 2 School of Economics and Management, Jiangxi Normal University, Nanchang 330022, China; 202540100885@jxnu.edu.cn; 3 Jiangxi Provincial Key Laboratory of Functional Molecular Materials Chemistry, Jiangxi University of Science and Technology, Ganzhou 341000, China; 4 Jiangxi Provincial Engineering Research Center of Blockchain Data Security and Governance, Nanchang 330031, China

**Keywords:** multimodal representation learning, graph neural networks, cross-modal fusion, information bottleneck theory

## Abstract

Accurate matching between molecular structures and NMR spectra is an important task in automated structure elucidation. However, existing methods still face difficulties in jointly modeling multi-scale molecular topology and effectively exploiting the complementary information provided by paired ^1^H and ^13^C NMR spectra. To address these limitations, we propose SpecMol-MatchNet, a multimodal matching framework that integrates a hybrid molecular graph encoder, branch-specific spectral feature learning, and residual multimodal fusion. In the molecular branch, attention-based graph interaction is combined with multi-scale neighborhood aggregation to capture structural cues at different receptive fields. In the spectral branch, branch-specific attention enhancement and joint gating are introduced to better exploit the complementary characteristics of paired ^1^H and ^13^C spectra. The resulting molecular and spectral representations are integrated through a residual fusion module for final matching prediction. Experimental results on benchmark datasets demonstrate that SpecMol-MatchNet achieves consistently better overall performance than representative baseline methods.

## 1. Introduction

Molecular structure elucidation occupies a central position in chemical and materials research, as it is essential for ensuring the reliability of synthetic outcomes and for enabling the discovery of new molecules [1]. Among various structural characterization techniques, nuclear magnetic resonance (NMR) spectroscopy is one of the most powerful tools, providing rich information about atomic environments, functional-group types, and molecular topology. Consequently, NMR is widely used in organic synthesis, natural product research, and drug discovery. However, in complex reaction systems, NMR spectral interpretation is often hindered by signal overlap, noise, and baseline drift. Manual analysis is time-consuming and labor-intensive, relies heavily on expert knowledge, and is increasingly inadequate for high-throughput and automated experimentation.

To alleviate these limitations, researchers have long sought computational approaches to assist NMR-based structure elucidation. Early efforts led to the development of computer-assisted structure elucidation (CASE) systems, which aim to automate the interpretation process. Nevertheless, these systems typically require substantial human intervention and often struggle with complex molecular structures [2,3].

With the rapid development of artificial intelligence, deep learning methods have recently been introduced into NMR data analysis. Some research focuses on predicting molecular structures from a single spectrum, for instance, by employing neural networks to infer structures directly from ^1^H or ^13^C spectra, or by using multitask learning frameworks to predict molecular attributes from one-dimensional (1D) NMR data [4,5,6]. Another line of work aims to improve the accuracy of chemical-shift prediction by combining quantum-chemical calculations with machine learning, thereby generating more reliable spectral simulations [7]. While these approaches have significantly advanced automated structure elucidation, they largely rely on single-modality information, which may lead to ambiguities when dealing with complex reaction systems.

Recently, representation learning has emerged as a key paradigm for modeling heterogeneous scientific data. In particular, multimodal representation learning enables the integration of complementary information from different data sources, allowing models to capture richer relationships across modalities [8]. Motivated by this idea, recent studies have explored multimodal fusion strategies that integrate molecular structural priors with NMR spectral features to enhance analysis performance [9,10]. For example, some works have proposed matching frameworks that combine molecular graphs with spectral images to determine whether a spectrum corresponds to a candidate molecule, demonstrating promising results in applications such as reaction monitoring [11]. Despite these advances, current multimodal graph–spectrum matching methods still face several limitations. First, existing molecular encoders often struggle to simultaneously capture local bonded environments, intermediate neighborhood context, and broader topological dependencies in a unified manner, which may restrict the discriminative ability of molecular representations for challenging matching tasks. Second, although ^1^H and ^13^C NMR spectra provide complementary information, their differences in peak density, chemical-shift distribution, and feature saliency are not always sufficiently modeled by shared feature extraction or shallow fusion strategies. Third, many existing methods rely on direct concatenation for multimodal integration, which may limit the effective utilization of complementary structural and spectral cues.

To address these limitations, we propose SpecMol-MatchNet, a multimodal matching framework with targeted designs in both the molecular and spectral branches. In the molecular branch, we combine attention-based graph interaction with multi-scale neighborhood aggregation to encode structural information at different receptive fields. In the spectral branch, we employ branch-specific attention enhancement and joint gating to better exploit the complementarity between paired ^1^H and ^13^C spectra. The resulting molecular and spectral representations are then integrated through a residual fusion module for final matching prediction. Experimental results on benchmark datasets demonstrate that the proposed framework achieves better overall performance than representative baselines under the evaluated matching settings.

The main contributions of this work are summarized as follows:

(1) Hybrid molecular representation learning. We design a hybrid molecular encoder that combines attention-based graph interaction with multi-scale neighborhood aggregation to capture complementary structural cues at different receptive fields for molecule–spectrum matching.
(2) Cross-spectral complementary modeling. We develop a spectral modeling strategy for paired ^1^H and ^13^C NMR spectra using branch-specific attention enhancement and joint gating, enabling more effective utilization of cross-spectrum complementarity.
(3) Residual multimodal fusion and empirical validation. We integrate molecular and spectral representations through a residual fusion design and validate the proposed framework through comparative and ablation experiments, showing improved overall matching performance on benchmark datasets.

## 2. Related Work

In recent years, the growing adoption of deep learning in chemical and molecular analysis has substantially advanced NMR-assisted molecular characterization. Along the unimodal modeling line, researchers mainly focus on learning from either spectral data or molecular structures alone to accomplish structure-related tasks. Cortés et al. [12] reviewed recent progress in machine learning for computational NMR-aided structural elucidation, showing that data-driven methods have become increasingly important in assisting spectrum interpretation and molecular identification. In addition, Wei et al. [11] proposed a deep learning-based method for compound identification in NMR spectra of mixtures, demonstrating the potential of learning directly from spectral observations in complex mixture settings. On the molecular side, Li et al. [13] systematically summarized deep learning methods for molecular representation and property prediction, highlighting the importance of graph-based structural modeling in chemistry. Although these studies have promoted the automation of NMR analysis and molecular modeling, they still rely primarily on a single information source and therefore may have limited ability to resolve ambiguous molecule–spectrum associations in complex settings.

Because unimodal methods rely on a single data dimension, they cannot fully exploit the complementarity between molecular structures and spectral observations. Consequently, multimodal matching and fusion strategies have emerged as an important research direction. Tian et al. [14] developed a deep learning model for NMR spectra image matching to target compounds in chemical reaction monitoring, showing that joint modeling of spectral images and candidate compounds can effectively improve compound identification. This line of work demonstrates the promise of structure–spectrum matching for molecular analysis.

At the same time, broader multimodal and multi-view learning studies have provided useful methodological insights for integrating heterogeneous data. Multimodal learning research has emphasized that heterogeneous inputs often require dedicated strategies for data integration, feature engineering, and scalable model learning [15,16]. Meanwhile, multi-view learning studies show that different views typically contain both shared and complementary information, while also introducing redundancy, which makes effective fusion nontrivial. Representative fusion strategies include early fusion, late fusion, hybrid fusion, and score fusion, and effective modeling often requires balancing view-specific representation learning with cross-view interaction [17]. In addition, graph-based multi-view contrastive learning has explored incorporating graph structure into multi-view representation learning to improve consistency and discrimination across views [18]. These insights are highly relevant to molecular graph–spectrum matching, where molecular structures and paired ^1^H/^13^C NMR spectra provide different but related descriptions of the same compound. However, existing graph–spectrum matching methods still show limitations in sufficiently modeling multi-scale molecular topology, in exploiting the complementary characteristics of paired ^1^H and ^13^C spectra, and in designing effective multimodal interaction strategies. Motivated by these gaps, our work formulates molecule–spectrum association as a multimodal matching problem and introduces targeted designs in both the molecular and spectral branches, together with residual fusion for final prediction.

## 3. Method

### 3.1. Task Definition

Given a candidate molecule *m* (represented as a molecular graph G=(V,E)) and its corresponding pair of one-dimensional NMR spectral images (^1^H and ^13^C), the objective is to determine whether the molecule and the spectra correspond. Specifically, the goal is to output a binary classification label y∈{0,1}.

### 3.2. Overall Framework

**Figure 1:**
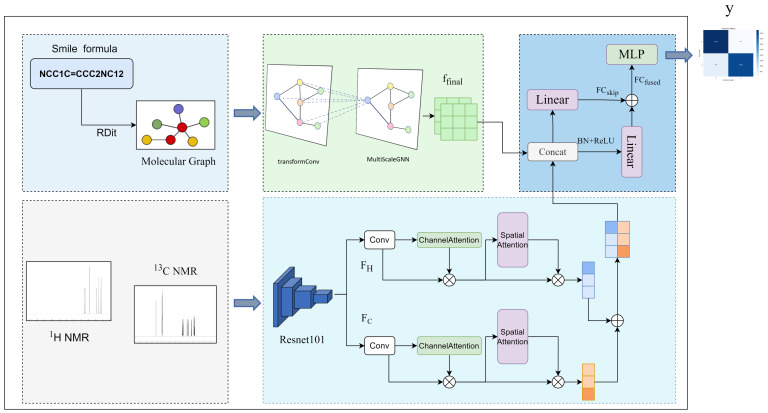
Overall architecture diagram. The model integrates molecular graph encoding, paired ^1^H/^13^C spectral feature extraction, and residual multimodal fusion for molecule–spectrum matching.

The overall framework comprises two primary modules: a molecular feature extraction submodule and an NMR spectral image submodule, followed by multimodal feature fusion for prediction. The molecular branch models atomic topological relations, while the spectral branch focuses on peak morphology and chemical-shift regions. The two representations are integrated through a residual fusion module, and a multilayer perceptron classifier ultimately outputs the binary class probability. The overall architecture is illustrated in Figure 1.

### 3.3. Molecular Feature Extraction

**Figure 2:**
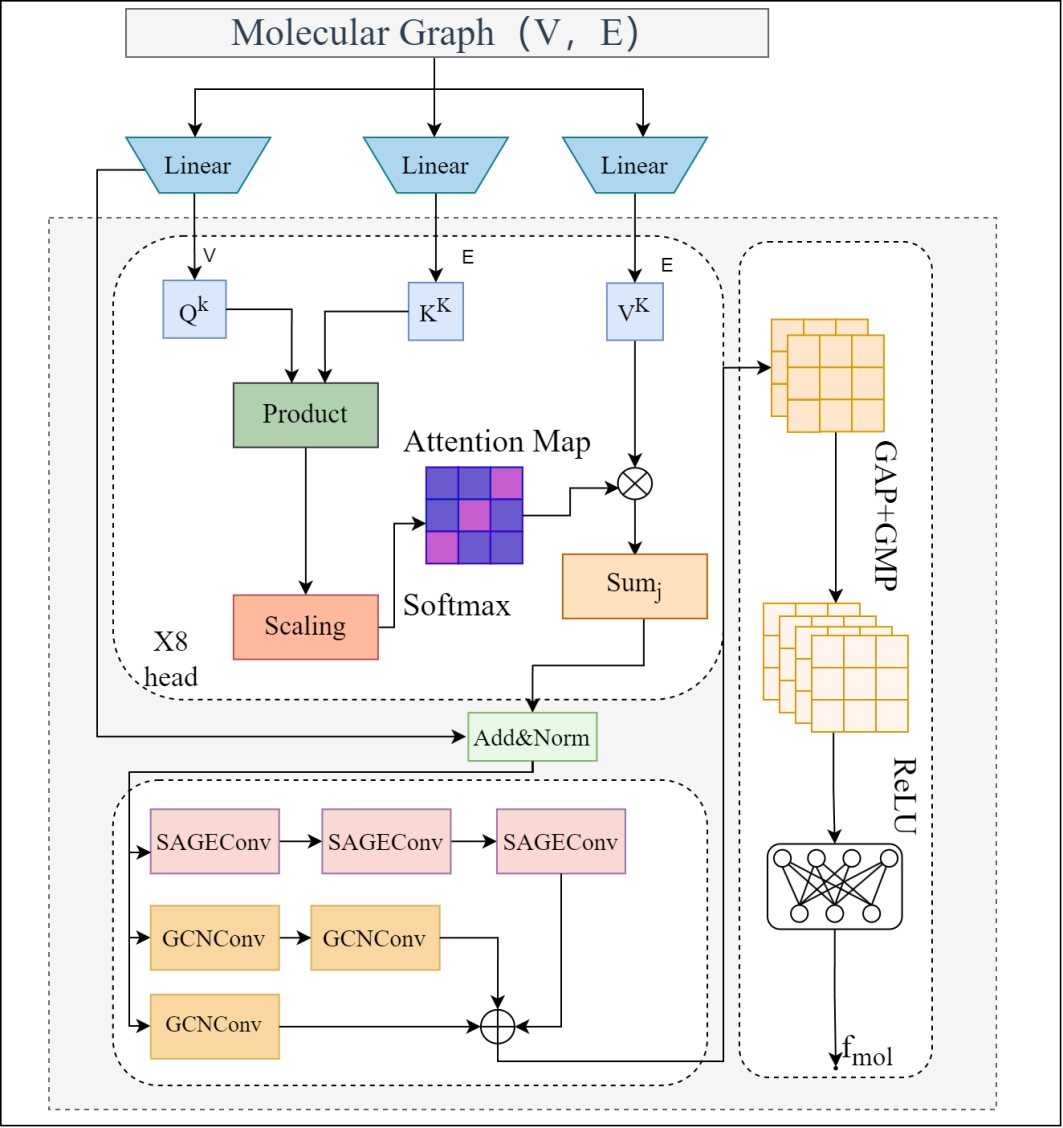
Molecular feature extraction structure diagram. A hybrid graph encoder combines TransformerConv with multi-scale graph aggregation to capture structural information at different receptive fields.

In the molecular structure submodule, we employ a graph neural network (GNN)-based feature extraction approach [19] to model the topological connectivity among atoms within the molecule. The architecture of the molecular feature extraction module is shown in Figure 2.

The specific architecture is as follows. The molecular structure is first represented as a graph G=(V,E), where the node set V denotes the atoms in the molecule. For each node $v_{i}\in V$, we extract its corresponding atomic feature vector $x_{i}\in R^{d\times |V|}$, thereby forming the node feature matrix X, where |V| is the number of atoms and *d* is the dimensionality of the initial atomic features. The edge set E defines the graph topology and is used in subsequent graph convolution and attention computations to determine the information flow pathways between nodes. To capture both broader dependencies and local topological relations, we adopt a hybrid architecture that combines attention-based graph convolution with a Multi-Scale Graph Neural Network (MSGNN). Specifically, TransformerConv [20] with sparse self-attention is employed to strengthen graph interaction, followed by a three-branch multi-scale GNN that fuses neighborhood information with different receptive fields. A dual global pooling operation is then applied to obtain a fixed-length molecule-level representation, which is further transformed by a two-layer multilayer perceptron to yield a compact feature vector.

First, by projecting the node feature matrix X into multiple attention heads, we compute query (Q), key (K), and value (V) vectors. Subsequently, a linear mapping is applied to the node feature matrix for each attention head to obtain Q, K, and V as follows:

(1) $$Q^{\left(k\right)}=XW_{Q}^{\left(k\right)},\;K^{\left(k\right)}=XW_{K}^{\left(k\right)},\;V^{\left(k\right)}=XW_{V}^{\left(k\right)}$$

where $Q^{\left(k\right)}$, $K^{\left(k\right)}$, and $V^{\left(k\right)}$ denote the query, key, and value matrices of the *k*-th attention head, respectively, and $W_{Q}^{\left(k\right)}$, $W_{K}^{\left(k\right)}$, and $W_{V}^{\left(k\right)}$ are the learnable projection matrices that map node features into the query, key, and value spaces. These projections evenly partition the original *d* channels across heads, thereby introducing cross-head representational diversity while keeping the overall computational budget controlled. Meanwhile, scaled dot-product attention is employed to compute message weights and their aggregation within the adjacency, thereby enforcing the constraints induced by the graph’s edge set. The specific formulas are as follows:

(2) $${\mathrm{Attn}}_{ij}^{\left(k\right)}=\mathrm{softmax}\left(\frac{Q^{\left(k\right)}K^{{\left(k\right)}^{T}}}{\sqrt{d_{k}}}\right)$$

Here, $Q^{\left(k\right)}K^{{\left(k\right)}^{T}}$ denotes the dot product between the query and key representations in the *k*-th attention head, and $\sqrt{d_{k}}$ is the per-head dimensionality used to scale the raw attention scores. The inter-node attention scores are then normalized with a softmax over each node’s neighborhood to yield the final weight distribution. Finally, the neighbor value features $V_{j}^{\left(k\right)}$ are combined via a weighted aggregation according to these attention weights to produce the attention-aggregated output for each node:

(3) $$H_{i}^{\left(k\right)}=\sum_{j\in N(i)}{\mathrm{Attn}}_{ij}^{\left(k\right)}V_{j}^{\left(k\right)}$$

where $H_{i}^{\left(k\right)}$ denotes the aggregated feature of the *k*-th attention head, with each head capturing distinct attention relationships. Finally, the eight heads are concatenated along the channel dimension to form a shared structure-aware representation:

(4) $$H^{\left(0\right)}{=∥}_{k=1}^{8}H^{\left(k\right)}$$

Here, $H^{\left(0\right)}$ denotes the concatenated representation that serves as the shared node features. Building on $H^{\left(0\right)}$, we construct three graph branches with progressively enlarged receptive fields to model local, intermediate, and broader topological context. For the short- and mid-range branches, we adopt the Graph Convolutional Network (GCN) [21] for feature aggregation. Since GCN updates node representations by aggregating features from each node and its neighbors, a single-layer GCN is used to emphasize local atom–bond environments, while a deeper two-layer GCN is used to capture broader neighborhood context. The specific formulas are as follows:

(5) $$H_{\mathrm{short}}=\phi \left({\mathrm{GCN}}^{\left(0\right)}\left(H^{\left(0\right)}\right)\right)$$

where ϕ denotes the ReLU activation function. This branch emphasizes aggregation within the local atom–bond environment and is used to characterize fine-grained local structural cues. Furthermore, building upon the short-range branch, we expand the receptive field by one hop and employ a two-layer GCN to hierarchically aggregate contextual information from second-order neighbors:

(6) $$H_{\mathrm{mid}}=\phi \left({\mathrm{GCN}}^{\left(2\right)}\left(\phi \left({\mathrm{GCN}}^{\left(1\right)}\left(H^{\left(0\right)}\right)\right)\right)\right)$$

The mid-range branch employs a two-layer stack, which extends the receptive field to approximately two hops and provides broader neighborhood context for subsequent long-range modeling. Subsequently, to cover a broader structural scope, we adopt GraphSAGE [22] for multi-hop aggregation, leveraging learnable aggregators to characterize longer-range topological dependencies spanning multiple hops. The specific formulas are as follows:

(7) $$H_{\mathrm{long}}={\mathrm{SAGE}}^{\left(3\right)}\left({\mathrm{SAGE}}^{\left(2\right)}\left({\mathrm{SAGE}}^{\left(1\right)}\left(H^{\left(0\right)}\right)\right)\right)$$

Finally, we concatenate the representations from the three scales along the channel dimension and use a linear fusion layer for alignment and reweighting:

(8) $$H_{\mathrm{out}}=W\cdot [H_{\mathrm{short}}\left|\right|H_{\mathrm{mid}}\left|\right|H_{\mathrm{long}}]+b$$

where || denotes feature concatenation, and W and b are the weight matrix and bias of the linear transformation used to map the concatenated features into the final output space. This fusion combines structural cues from local, intermediate, and broader graph contexts, thereby yielding an informative node representation for the final feature fusion. Subsequently, to obtain a fixed-length molecule-level vector while retaining both salient activation information and overall distributional statistics, we apply global max pooling and global average pooling to the node representations $H_{\mathrm{out}}$ and concatenate the results, as given by the following:

(9) $$f_{\mathrm{mol}}=\mathrm{concat}\left(\mathrm{GMP}\left(H_{\mathrm{out}}\right),\mathrm{GAP}\left(H_{\mathrm{out}}\right)\right)$$

Specifically, GMP takes the per-dimension maximum over the node feature matrix to capture salient activation information, whereas GAP computes the per-dimension mean to characterize global distributional properties. The two outputs are then concatenated to obtain the molecule-level representation $f_{\mathrm{mol}}$, providing a molecular structural descriptor for subsequent feature fusion. Finally, this module performs dimensionality reduction using two fully connected layers:

(10) $$f_{\mathrm{final}}={\mathrm{FC}}_{128}\left({\mathrm{FC}}_{1500}\left(f_{\mathrm{mol}}\right)\right)$$

The first fully connected layer increases the feature dimensionality, using ReLU activation and Dropout regularization to enhance representational capacity while reducing overfitting risk. The second fully connected layer then compresses the dimensionality to yield a compact representation. The module ultimately outputs a fixed-dimensional molecular graph feature vector $f_{\mathrm{final}}$, which can be directly used for downstream multimodal feature fusion and matching/prediction tasks.

### 3.4. NMR Spectral Image Feature Extraction

**Figure 3:**
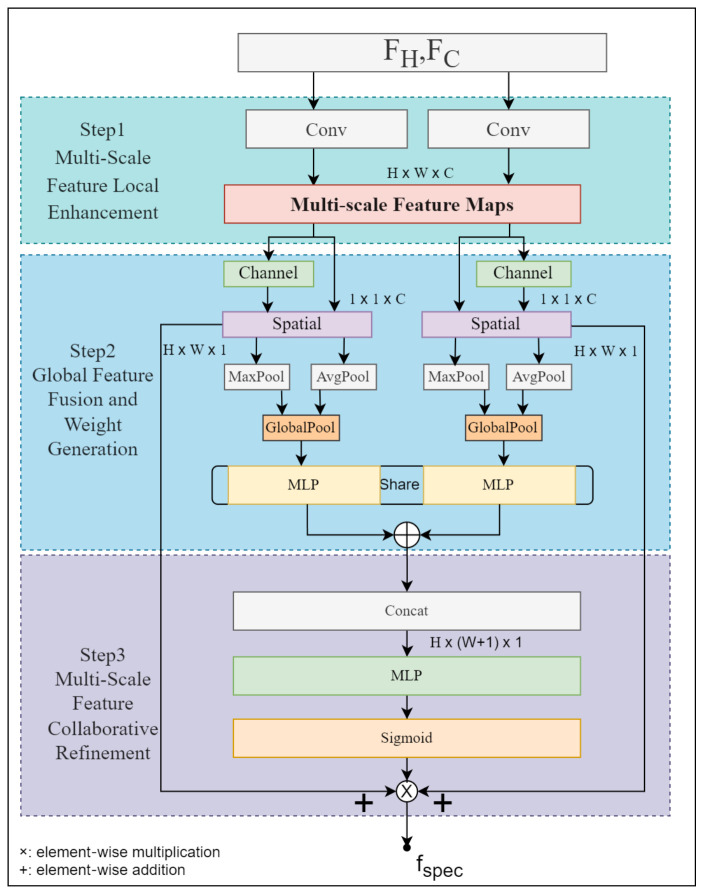
Multi-Scale Convolutional Attention Module. This module enhances spectral features through multi-scale convolution, channel–spatial attention, and cross-branch interaction between ^1^H and ^13^C spectra.

To enhance the discriminative power of spectral peaks and key chemical shifts without introducing additional training instability, we employ a ResNet101 with frozen parameters as a shared backbone to extract high-level features from both ^1^H and ^13^C spectra. Since the two spectrum types differ in peak density and chemical-shift distribution, a multi-scale convolutional attention module is used for branch-specific enhancement, followed by cross-branch gating for collaborative recalibration. Within this module, the two feature maps are respectively processed by distinct Convolutional Block Attention Modules (CBAMs), enabling differential feature extraction tailored to the two NMR spectra. The architecture of the multi-scale convolutional attention module is shown in Figure 3.

Specifically, we first apply convolutional operations to the two backbone outputs, $F_{H}$ and $F_{C}$, to align the channel dimensions of the two branches and control the subsequent computational cost:

(11) $${\hat{F}}_{i}={\mathrm{Conv}}_{1\times 1}\left(F_{i}\right)$$

${\mathrm{Conv}}_{1\times 1}$ denotes a 1×1 convolutional layer that compresses the 2048-dimensional features output by ResNet101 to 16 dimensions. This operation acts solely on the channel dimension and leaves the spatial resolution unchanged, thereby allowing both feature streams to enter the multi-scale convolutional attention module and the subsequent cross-branch gating with a unified number of channels. Within each branch, the multi-scale convolutional attention module enhances salient features through a cascaded channel–spatial attention structure. Specifically, for each spectral feature map ${\hat{F}}_{i}$, spatial information is aggregated using global max pooling (GMP) and global average pooling (GAP); the resulting descriptors are passed through shared network layers and fused via element-wise addition to produce the channel-attention weight map. The computation is given by the following:

(12) $$M_{c}=\sigma \left(\mathrm{MLP}\left(\mathrm{GAP}\left({\hat{F}}_{i}\right)\right)+\mathrm{MLP}\left(\mathrm{GMP}\left({\hat{F}}_{i}\right)\right)\right)$$

Here, ${\hat{F}}_{i}$ denotes the input feature map; MLP denotes a shared two-layer multilayer perceptron; σ denotes the Sigmoid activation function; and $M_{c}\in {\left(0,1\right)}^{C}$ denotes the channel-attention weights used to emphasize salient channel features. In contrast to channel attention, the spatial-attention submodule places greater emphasis on the importance of different locations in NMR spectral images. Specifically, global max pooling and global average pooling are applied to the feature map refined by channel attention to obtain two spatial descriptor maps; these are concatenated and passed through the MLP followed by a Sigmoid to produce a two-dimensional spatial attention map, which is then used as a mask and applied to the features via element-wise multiplication to highlight critical regions. The main computations are as follows:

(13) $$\begin{array}{cc} M_{s}\left(M_{c}\right) & =\sigma \left(f^{k\times k}\left([\mathrm{AvgPool}\left(M_{c}\right),\mathrm{MaxPool}\left(M_{c}\right)]\right)\right) \end{array}$$

(14) $$F_{b}^{\prime }=M_{s}\left(M_{c}\right)⊗M_{c}$$

Here, ${\mathrm{Avg}}_{c}$ and ${\mathrm{Max}}_{c}$ denote the channel-wise average and maximum operations, respectively; ${\mathrm{Conv}}_{7\times 7}$ denotes a convolutional layer; $M_{s}\in {\left(0,1\right)}^{7\times 7}$ is the spatial attention mask; and ⊗ denotes element-wise multiplication. The final output is the spatial-attention-refined feature map $F_{b}^{\prime }$, which serves to highlight key regions in NMR spectral images.

Meanwhile, to exploit the complementarity between ^1^H and ^13^C spectra, a cross-branch joint gating strategy is applied after obtaining the two-dimensional spatial attention map. Specifically, the two spectral feature streams are first globally pooled to form global descriptors; the descriptors are then concatenated and fed into the shared MLP to compute the joint channel weights:

(15) $$f_{\mathrm{spec}}=\sigma \left(\mathrm{MLP}\left(\mathrm{Concat}\left(g_{H},g_{C}\right)\right)\right)$$

Here, $g_{H}$ and $g_{C}$ denote the globally pooled features of the ^1^H and ^13^C spectra after attention processing, respectively, and σ denotes the Sigmoid activation function. The resulting weights are then partitioned by branch and fed back to the corresponding feature maps, achieving channel-level recalibration jointly determined by both branches. Finally, adaptive global average pooling is applied to both feature streams, and the pooled vectors are concatenated to facilitate subsequent prediction.

### 3.5. Feature Fusion

The molecular feature vector $f_{\mathrm{final}}$ and the spectral feature vector $f_{\mathrm{spec}}$ are concatenated to obtain a multimodal joint representation. To facilitate feature interaction while maintaining a lightweight integration pathway, this module implements feature fusion through a residual connection mechanism comprising a main-branch transformation and a skip-connection fusion. Specifically, the joint representation undergoes a two-layer nonlinear transformation in the main branch and is then summed with the linear skip branch in the same dimensional space to complete the fusion. The specific formulation is as follows:

(16) $$F_{\mathrm{fused}}={\mathrm{FC}}_{2}\left(\mathrm{BN}\left(\phi \left({\mathrm{FC}}_{1}\left(u\right)\right)\right)\right)+{\mathrm{FC}}_{\mathrm{skip}}\left(u\right)$$

where $u=[f_{\mathrm{final}};f_{\mathrm{spec}}]$ denotes the multimodal joint representation; ${\mathrm{FC}}_{1}$ and ${\mathrm{FC}}_{2}$ denote the fully connected transformations in the main branch; ${\mathrm{FC}}_{\mathrm{skip}}$ denotes the skip connection implemented via linear projection; and $F_{\mathrm{fused}}$ denotes the unified representation after residual fusion. Subsequently, the fused representation is fed into a three-layer fully connected multilayer perceptron, whose principal computations are given as follows:

(17) $$\hat{y}=\sigma \left({\mathrm{FC}}_{\mathrm{out}}\left(\mathrm{BN}\left(\phi \left({\mathrm{FC}}_{\mathrm{pred}}\left(F_{\mathrm{fused}}\right)\right)\right)\right)\right)$$

where ${\mathrm{FC}}_{\mathrm{pred}}$ and ${\mathrm{FC}}_{\mathrm{out}}$ denote the fully connected mappings of the multilayer perceptron; ϕ represents an activation function (e.g., ReLU), BN denotes Batch Normalization, and $F_{\mathrm{fused}}$ is the multimodal feature vector obtained from the fusion module. $\hat{y}\in \left(0,1\right)$ denotes the final predicted probability used for subsequent binary classification decisions to determine whether the molecule and its NMR spectra correspond.

## 4. Experiment

### 4.1. Datasets

This study was conducted on public datasets sourced from PubChem and the GDB13 database. After screening with RDKit, we selected 50,761 small- to medium-sized molecules containing only C, N, O, and H atoms, with the number of non-hydrogen atoms not exceeding 16. The dataset includes paired ^1^H NMR and ^13^C NMR spectral images. The ^1^H spectra were acquired at 500.12 MHz over the range of 2 to 12.05 ppm, and the ^13^C spectra were acquired at 125.03 MHz over the range of 2 to 230 ppm in CDCl_3_, yielding a total of 101,522 spectral images.

Positive and negative molecule–spectrum pairs were constructed at a ratio of 1:1, and the complete dataset was divided into training, validation, and test sets at a ratio of 8:1:1. For evaluation, two test subsets, namely Test_rand and Test_diff, were used. Test_rand was constructed by randomly sampling matched and mismatched molecule–spectrum pairs, and was used to evaluate the overall matching performance of the model under a general random setting. In contrast, the negative samples in Test_diff were selected from compounds with relatively large structural differences from the target molecule, and this subset was used to evaluate the discriminative ability of the model when handling molecules with larger structural differences.

### 4.2. Evaluation Metrics

This study evaluates the performance of SpecMol-MatchNet on the NMR spectrum–molecular structure matching task. Precision, Recall, and F1-Score are used as auxiliary metrics, while Accuracy and the area under the ROC curve (AUC) serve as the primary evaluation criteria [23,24]. Accuracy reflects the proportion of correctly classified samples among all predictions, directly measuring the model’s overall performance on the molecule–spectrum matching task. It is computed as follows:

(18) $$\mathrm{Accuracy}=\frac{\mathrm{TP}+\mathrm{TN}}{\mathrm{TP}+\mathrm{TN}+\mathrm{FP}+\mathrm{FN}}$$

Here, TP (True Positive) denotes the number of positive samples (molecule–spectrum matches) correctly predicted as positive; TN (True Negative) denotes the number of negative samples (molecule–spectrum non-matches) correctly predicted as negative; FP (False Positive) denotes the number of negative samples incorrectly predicted as positive; and FN (False Negative) denotes the number of positive samples incorrectly predicted as negative.

### 4.3. Implementation Details

To ensure fairness and reproducibility, all models were trained under the same data split and optimization protocol. The dataset was divided into training, validation, and test sets at a ratio of 8:1:1. A fixed random seed was used throughout all experiments. The Adam optimizer was employed with an initial learning rate of $1\times 10^{-4}$, and the loss function was binary cross-entropy (BCELoss). The batch size was set to 32, and training was conducted for 100 epochs. Images across all datasets were uniformly resized to 256×256 pixels. For each model, the checkpoint achieving the highest Accuracy on the validation set was selected as the best checkpoint and subsequently evaluated on the test set. Unless otherwise stated, the same training settings were used for all compared methods. All experiments were implemented with CUDA 11.8, PyTorch 2.0.0, and Python 3.8, and were conducted on a platform equipped with All experiments were implemented with CUDA 11.8, PyTorch 2.0.0, and Python 3.8, and were conducted on a server equipped with 12 vCPU Intel(R) Xeon(R) Platinum 8352V CPUs (Intel Corporation, Santa Clara, CA, USA) and four NVIDIA GeForce RTX 4090 GPUs (NVIDIA Corporation, Santa Clara, CA, USA).

### 4.4. Baseline Models

To evaluate the performance of the proposed SpecMol-MatchNet, we selected five representative baseline models for comparison. Specifically, GCN + ResNet-101 and GAT + ResNet-101 were used as straightforward bimodal baselines, where the graph branch models molecular topology and ResNet-101 encodes spectral image features. In addition, ResNet-50, DenseNet, and EfficientNet were included as representative single-branch image-based baselines to assess the spectral representation capability without explicit graph modeling.

### 4.5. Compare Experiment

Table 1 presents the comparative results of all models on the two test sets. Overall, SpecMol-MatchNet achieves the best results across most evaluation metrics on both Test_diff and Test_rand. On Test_diff, the proposed model attains an AUC of 0.99, together with a precision of 0.99, a recall of 0.89, and an F1 score of 0.94, indicating improved discrimination between matched and mismatched molecule–spectrum pairs under this more challenging setting.

By contrast, although several baselines, especially GCN + ResNet-101 and EfficientNet, achieve competitive precision, their recall values are generally lower. In particular, GCN + ResNet-101 and ResNet-50 maintain relatively high precision but exhibit reduced recall, suggesting that they are more likely to miss matched pairs when structural variation becomes larger. This difference is more noticeable on Test_diff, where the proposed model shows a better balance between precision and recall.

On Test_rand, SpecMol-MatchNet achieves an AUC of 0.92, a precision of 0.85, a recall of 0.89, an F1 score of 0.87, and an accuracy of 0.86. Compared with the baseline models, the proposed framework still achieves the best overall results under the random pairing setting. In particular, its relatively higher recall suggests improved ability to identify true matched pairs in the presence of increased randomness.

Overall, these results indicate that integrating molecular graph features with paired NMR spectral features is beneficial for molecule–spectrum matching. Although the performance gains over the strongest baselines are moderate on some settings, the proposed model consistently achieves the best overall results across the evaluated metrics.

### 4.6. Ablation Experiment

To further assess the effectiveness of the components in SpecMol-MatchNet, we conducted ablation studies by progressively simplifying the graph and spectral branches and evaluating the resulting performance changes. Specifically, we considered four representative variants: SingleGCN_CBAM, which removes the multi-scale graph modeling and uses standard CBAM in the spectral branch; MultiScaleGNN_MultiScaleCBAMBlock, which restores multi-scale graph modeling and multi-scale spectral attention but does not use TransformerConv; MultiScaleGNN_TransformerConv_CBAMBlock, which further introduces TransformerConv while using standard CBAM in the spectral branch; and SingleGCN_MultiScaleCBAMBlock_TransformerConv, which keeps a simplified graph backbone while combining TransformerConv with the MultiScaleCBAMBlock. The complete model is denoted as all. This design allows us to separately examine the contributions of multi-scale graph modeling, Transformer-based graph interaction, and multi-scale spectral attention.

**Figure 4:**
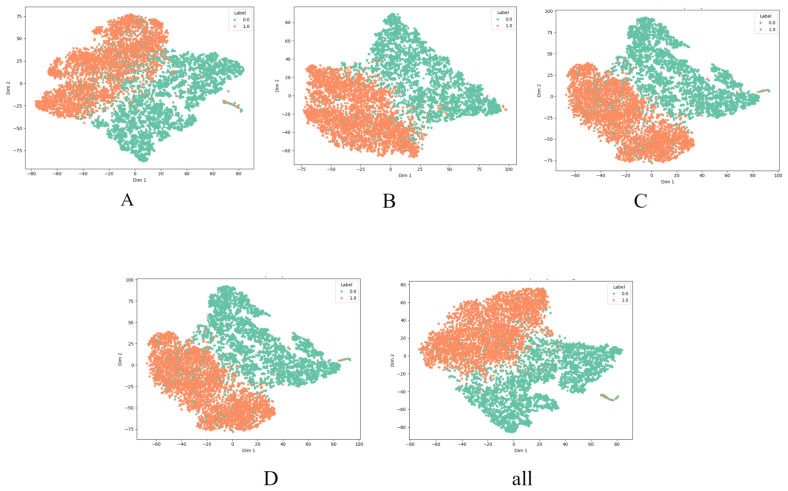
T-SNE visualization under different ablation configurations.

The impact of each module on feature discriminability can also be visually examined through the t-SNE visualization of pre-output-layer features under different ablation configurations, as shown in Figure 4.

For ease of presentation, SingleGCN_CBAM, MultiScaleGNN_MultiScaleCBAMBlock, MultiScaleGNN_TransformerConv_CBAMBlock, and SingleGCN_MultiScaleCBAMBlock_ TransformerConv are denoted as Models A, B, C, and D, respectively.

As shown in Table 2, the most substantial performance drop occurs in Model A. On Test_diff, its AUC, Recall, and F1 decrease to 0.921, 0.518, and 0.667, respectively, while on Test_rand the corresponding values fall to 0.753, 0.602, and 0.603. These results suggest that simplifying both the graph branch and the spectral attention branch substantially weakens the model’s ability to distinguish matched from mismatched molecule–spectrum pairs, especially in the more challenging Test_diff setting.

Compared with Model A, Models B, C, and D progressively restore different components in the graph and spectral branches. Model B improves the results to 0.958 AUC and 0.802 F1 on Test_diff, and 0.832 AUC and 0.723 F1 on Test_rand, indicating that introducing multi-scale graph modeling and multi-scale spectral attention leads to a clear performance gain over the simplest variant. Model C further improves the results to 0.981 AUC, 0.928 F1, and 0.931 Accuracy on Test_diff, and 0.912 AUC, 0.852 F1, and 0.851 Accuracy on Test_rand. These results support the benefit of combining Transformer-based graph interaction with multi-scale graph aggregation for molecular structure modeling. Model D achieves 0.982 AUC, 0.931 F1, and 0.932 Accuracy on Test_diff, as well as 0.919 AUC, 0.873 F1, and 0.861 Accuracy on Test_rand, showing that even with a simplified graph backbone, reintroducing TransformerConv together with the MultiScaleCBAMBlock yields strong overall performance.

The full model achieves the best overall results, reaching 0.989 AUC, 0.939 F1, and 0.941 Accuracy on Test_diff, as well as 0.922 AUC, 0.871 F1, and 0.863 Accuracy on Test_rand. Although the margin over Models C and D is relatively modest, the full model consistently attains the strongest or near-strongest results across the evaluated settings, suggesting that the complete combination of graph-side and spectral-side designs provides the most effective overall configuration.

To further examine the computational cost of these variants, we additionally compared their parameter size, inference time, and memory usage, as summarized in Table 3. The results show that the frozen ResNet-101 backbone consistently contributes 42.50 M parameters across all variants, while the main complexity differences arise from the trainable graph-side and multimodal components. Specifically, Model A contains 42.91 M parameters in total, Model B contains 43.75 M parameters, Model C contains 44.12 M parameters, and Model D contains 43.30 M parameters, while the full model contains 44.12 M parameters. Their peak GPU memory allocations remain within a narrow range of approximately 336–340 MB, and their inference times are of the same order of magnitude under the evaluated setting. These results suggest that the full model achieves improved matching performance with only a moderate increase in computational cost.

Overall, the ablation results support the contribution of both the graph-side and spectral-side designs in SpecMol-MatchNet. In particular, the results indicate that multi-scale graph modeling provides the dominant gain, while the MultiScaleCBAMBlock further improves performance with only limited additional computational cost.

All results are reported as mean ± standard deviation over multiple runs with different random seeds.

## 5. Conclusions

In this work, we proposed SpecMol-MatchNet, a multimodal framework for molecular graph and NMR spectrum matching. The framework integrates a molecular feature extraction branch based on attention-enhanced graph interaction and multi-scale graph aggregation, a spectral feature extraction branch for paired ^1^H and ^13^C NMR spectra, and a residual fusion module for multimodal prediction.

Experimental results on benchmark datasets show that the proposed method achieves better overall matching performance than representative baseline models. In addition, the ablation results support the contributions of the main components in both the molecular and spectral branches, indicating that the proposed design is effective for integrating structural and spectral information in the matching task.

Overall, the results suggest that targeted multimodal modeling is beneficial for molecular graph–spectrum alignment. In future work, we will further evaluate the framework under more challenging spectral conditions, compare it with stronger recent baselines, and investigate its efficiency and scalability in larger candidate libraries. 

## Figures and Tables

**Table 1:** Comparative performance of models on test sets.

Model	Test	AUC	Precision	Recall	F1	Accuracy
GCN + ResNet-101 [14]	Test_diff	0.98	0.96	0.82	0.88	0.89
	Test_rand	0.86	0.77	0.82	0.79	0.79
DenseNet [25]	Test_diff	0.96	0.93	0.81	0.86	0.87
	Test_rand	0.85	0.75	0.81	0.78	0.77
ResNet-50 [26]	Test_diff	0.97	0.93	**0.89**	0.91	0.92
	Test_rand	0.86	0.75	**0.89**	0.82	0.80
GAT + ResNet-101 [14]	Test_diff	**0.99**	0.98	0.87	0.92	0.93
	Test_rand	0.91	0.82	0.87	0.84	0.84
EfficientNet [27]	Test_diff	**0.99**	0.98	0.86	0.92	0.92
	Test_rand	0.90	0.81	0.86	0.83	0.83
Ours	Test_diff	**0.99**	**0.99**	**0.89**	**0.94**	**0.94**
	Test_rand	**0.92**	**0.85**	**0.89**	**0.87**	**0.86**

*Note:* Bold values indicate the best performance for each metric within the same test set. When multiple models achieve the same best value, all tied values are shown in bold.

**Table 2:** Ablation experiment results.

Model	Test	AUC	Precision	Recall	F1	Accuracy
A	Test_diff	0.921±0.011	0.923±0.011	0.518±0.019	0.667±0.015	0.742±0.013
	Test_rand	0.753±0.014	0.648±0.015	0.602±0.018	0.603±0.017	0.721±0.014
B	Test_diff	0.958±0.007	0.951±0.008	0.681±0.015	0.802±0.011	0.823±0.010
	Test_rand	0.832±0.012	0.768±0.013	0.683±0.014	0.723±0.013	0.743±0.012
C	Test_diff	0.981±0.005	0.979±0.005	0.882±0.009	0.928±0.006	0.931±0.006
	Test_rand	0.912±0.008	0.831±0.010	0.879±0.010	0.852±0.009	0.851±0.008
D	Test_diff	0.982±0.004	0.981±0.005	0.883±0.009	0.931±0.006	0.932±0.006
	Test_rand	0.919±0.008	0.849±0.009	0.881±0.009	0.873±0.008	0.861±0.008
all	Test_diff	0.989±0.002	0.991±0.004	0.891±0.006	0.939±0.004	0.941±0.004
	Test_rand	0.922±0.005	0.852±0.008	0.889±0.007	0.871±0.007	0.863±0.006

*Note:* Values are reported as mean ± standard deviation over five independent runs with different random seeds. Bold mean values indicate the best performance for each metric within the same test set.

**Table 3:** Computational cost comparison of different ablation variants.

Model	Total Params (M)	Time (ms/batch)	Peak Memory (MB)
A	42.913	25.97	335.57
B	43.748	30.29	338.26
C	44.115	23.78	339.66
D	43.300	19.77	337.31
all	44.116	21.22	339.67

## Data Availability

The data presented in this study are available on request from the corresponding author.
